# Conscientious commitment, professional obligations and abortion provision after the reversal of *Roe v Wade*

**DOI:** 10.1136/jme-2022-108731

**Published:** 2023-02-08

**Authors:** Alberto Giubilini, Udo Schuklenk, Francesca Minerva, Julian Savulescu

**Affiliations:** 1 Oxford Uehiro Centre for Practical Ethics and Wellcome Centre for Ethics and Humanities, University of Oxford, Oxford, UK; 2 Department of Philosophy, Queen's University, Kingston, Ontario, Canada; 3 Dipartimento di Filosofia Pietro Martinetti, University of Milan, Milano, Italy; 4 Centre for Biomedical Ethics, National University of Singapore, Singapore

**Keywords:** Conscientious Refusal to Treat, Abortion - Induced, Ethics- Medical

## Abstract

We argue that, in certain circumstances, doctors might be *professionally* justified to provide abortions even in those jurisdictions where abortion is illegal. That it is at least professionally permissible does not mean that they have an all-things-considered ethical justification or obligation to provide illegal abortions or that professional obligations or professional permissibility trump legal obligations. It rather means that professional organisations should respect and indeed protect doctors’ positive claims of conscience to provide abortions if they plausibly track what is in the best medical interests of their patients. It is the responsibility of state authorities to enforce the law, but it is the responsibility of professional organisations to uphold the highest standards of medical ethics, even when they conflict with the law. Whatever the legal sanctions in place, healthcare professionals should not be sanctioned by the professional bodies for providing abortions according to professional standards, even if illegally. Indeed, professional organisation should lobby to offer protection to such professionals. Our arguments have practical implications for what healthcare professionals and healthcare professional organisations may or should do in those jurisdictions that legally prohibit abortion, such as some US States after the reversal of *Roe v Wade*.

## Introduction

The reversal of the 1973 *Roe v Wade* ruling by the US Supreme Court in the 2022 *Dobbs v. Jackson Women’s Health Organization* 1 removed the Constitutional protection of women’s right to access abortion services in the USA. Individual States will have the authority to implement their own legislations about access to abortion services. Recent surveys suggest that 61% of US Americans think that ‘abortion should be legal in all or most cases’.^2^ However, different states will have different internal majorities that will result in uneven abortion legislations across the country. At the time of writing, eight states have introduced a ban on almost all abortions. Of these, five states (Alabama, Arkansas, Missouri, South Dakota and Texas) make no exception for pregnancies caused by incest or rape.^3^ It is hard to estimate how much support these laws enjoy in different states. A recent poll suggests that 60% of people in Texas support legal access to abortion, though the survey was funded by Planned Parenthood, with a sample of only 2000 people.^4^

Alongside the predictable public and political reactions, the ruling raises a number of ethical issues. One is, most obviously, the longstanding issue about women’s right to access abortion services, weighed against any moral and legal status of the fetus. Another one is whether access to abortion should be decided by elected representatives or should rather be a constitutional matter not subjected to a simple majority rule. These two issues have received and will presumably receive a lot of attention in public and academic discussion.

But the ruling also has implications for a third issue, namely that of professional obligations and permissible professional behaviour of healthcare providers. A legal ban on the provision of abortion services by healthcare professionals will not eliminate the medical need or the preference for access to safe abortion services. The WHO describes in a 2021 briefing paper unsafe abortions as the leading cause of maternal deaths and morbidities^5^ Often, backstreet abortions are performed in unsafe and unhygienic environments, sometimes by people who are not medically qualified and who cannot deal with medical complications that could arise. Partly for this reason, cases of ‘conscientious commitment’ by healthcare professionals to provide abortion services in contexts where it is illegal are well documented.^6^

Conscience is a faculty for moral judgements and moral action that includes a set of deeply held moral beliefs and values.^7^ Sometimes, conscience relates to beliefs on what is good for the patient. For example, someone might think that sterilisation or abortion is not in a patient’s best interest as they would prevent that patient from bearing children and raising a family. In the healthcare context, negative claims of conscience are claims by professionals to refuse to provide legally available medical services on the basis of personal moral, philosophical or religious beliefs. These cases have been extensively discussed in the literature on conscientious objection in healthcare. Less attention has been given to positive claims of conscience in the form of conscientious commitment. These are claims by professionals to provide services that are prohibited (either as a matter of law or policy), but that the professional conscientiously believes ought to be provided. While entailing the risk of severe legal consequences, conscientious commitment has historically been the start of legal challenges that paved the way to the decriminalisation of abortion in countries such as the UK and Canada.^8^

Conscientious commitment raises the question of when the reasons of conscience that support such provision are consistent with professional obligations or at least with what is professionally permissible. This is the issue we are going to explore in this article.

Most of the time, the type of healthcare individuals are legally entitled to receive coincides with what is considered good medical and medical ethics standards by the relevant professional bodies. However, professional and legal expectations sometimes come apart. The recent US Supreme Court decision is creating this situation in some US states. For example, the International Federation of Gynaecology and Obstetrics (FIGO) decried the US Supreme Court decision as a ‘catastrophic […] decision that will cost lives for years to come.’^9^ One might question FIGO’s claim or be more critical of the abortion ban. For the purpose of this paper, what matters is simply that there is a tension between the stance on abortion of certain professional organisations and the law. The question arises as to what professional obligations’ healthcare professionals are subjected to in such cases, and how professional organisations’ codes of practice should handle such cases.

At the moment, most non-sectarian (eg, non-religious) mainstream medical organisations in Western countries as well as the World Medical Association support the provision of abortion care.^10^ For example, the British Medical Association has a long-standing policy of supporting the 1967 UK Abortion Act which decriminalised abortion.^11^ The American Medical Association (AMA) states that ‘[t]he Principles of Medical Ethics of the AMA do not prohibit a physician from performing an abortion in accordance with good medical practice and under circumstances that do not violate the law’.^12^ The last statement by the AMA countenances the possibility that abortion is a part of good medical practice. The American College of Obstetrics and Gynaecology (ACOG) is more forthright by stating that ‘[a]bortion is an essential component of comprehensive medical care […]. It is unacceptable for doctors and healthcare professionals to be punished, fined, or sued and face imprisonment for delivering evidence-based care’.^13^

However, such professional codes of practice typically also include the explicit requirement that healthcare professionals operate within the law, as the AMA statement reported above exemplifies.

In this article, we will argue that, on a plausible account of professional responsibilities that we call ‘The Best Medical Interests’ account, healthcare professionals either retain the professional obligation to provide abortion that is illegal; or at the very least it is professionally permissible for them to do so, when it is otherwise endorsed by relevant professional organisations and when it is in the best medical interest of the woman.

Before getting to the core of this discussion, it is important to distinguish between ethical obligations, professional obligations and legal obligations.

Ethical obligations consist in what one ought to do, morally. Some of them are very general and arguably apply to everyone, such as the ethical obligation to abide by the law. However, ethical obligations are normally taken to be *prima facie* ones, that is, they are defeasible: if conflicting but stronger ethical obligations arise, the latter would prevail. For example, everyone has an ethical obligation to abide by the law, and also an ethical obligation to do what is just. If the latter trumps the former, then one has an all-things-considered ethical obligation to break unjust laws. When that is the case is, of course, up for debate. It depends, among other things, on whether we think there is an objective standard of morality that can be used to decide such cases.

Professional obligations are a specific type of ethical obligations that one has only *as a professional*. They are based on the ethical standards that apply specifically to the carrying out of activities within that profession. For example, healthcare professionals arguably have an ethical obligation to act in the best medical interest of their patients, but a random visitor at a hospital who is not a healthcare professional normally does not have that ethical obligation towards the patients in that hospital. Professional codes of practice typically establish professional obligations. Professionals are expected to follow them because there is a presumption that they reflect principles of good medical ethics arrived at through careful reflection and consideration of all the ethical issues involved—a presumption which, arguably, is not always correct.

Legal obligations are, most obviously, obligations as set out by the law, typically enforced through threats of punishments. For example, where abortion is illegal, providing abortions will likely result in fines or jailtime.

From these descriptions, it is easy to see how the three types of obligations are conceptually different. As such, they might either overlap or come into conflict with one another. If and when they do, different actors play different roles in addressing or solving these conflicts.

Enforcement of legal obligations and sanctions for non-compliance with them is up to the relevant authorities, like the police, courts, judges, public prosecutors. Adherence to ethical obligations is up to each individual’s assessment. Enforcement of professional obligations is up to the relevant professional bodies, who have the authority to sanction within the profession those who do not adhere to them. For example, they can warn, suspend or deregister individuals who acted unprofessionally or support those who acted professionally. Whether or not this also has legal consequences is not up to the professional organisation to decide or to sanction.

Thus, according to the account of professional obligations we are adopting, to say that healthcare professionals have a professional obligation to provide abortions where it is illegal—or at the very least to say that it is professionally permissible for them to do so—does not mean that they have an all-things-considered ethical obligation, nor that it is all-things-considered ethically permissible for healthcare professionals to break restrictive abortion laws.

Legal requirements trump professional requirements or whatever is taken to be professionally permissible. Doctors breaking the law should, *as citizens*, be prepared to pay the legal consequences. However, the task of professional organisations is to judge them *as professionals* and protect them when they act on professional standards, which include the pursuit of the patient’s best medical interests, as we are going to argue in the next sections.

### What are professional standards?

It is uncontroversial that to be a professional means, among other things, to be bound by obligations defined by professional standards. However, there is disagreement with regard to what falls within professional standards of healthcare and with regard to the appropriate procedure to determine them.

We can identify three possible ways to establish professional obligations. They are based on the contractual nature of professional roles and on standards of medical ethics. By entering the healthcare profession—exactly like any other profession—an individual enters into a contractual relationship with both society and the relevant professional bodies. The terms of the contract define professional standards and obligations. The following three models often overlap. This might make it difficult to see that they are conceptually different. However, their conceptual difference becomes apparent in cases of conflict, as might happen with certain restrictions on access to abortion.

#### The legal model of professional standards

According to the first criterion, professional standards are simply established by the legal framework in which the professional operates. This seems to follow from a contractualist view of professions and professional obligations. Typically, in a social contract, all parties make concessions to other parties involved in order to reach reasonable terms of cooperation and of reciprocity. There are different theories as to what constitutes reasonable contractual terms,^14 15^ but on any of those, contractual terms require one to act in ways that are not merely self-interested when relevant interests of other people are in tension with one’s own.

Entering a healthcare profession typically comes with monopolistic rights over the provision of certain services. This implies, among other things, that professionals’ right to practice healthcare is protected against unregulated competition. In most countries, performing medical procedures without the necessary qualifications that those societies formally recognise is a criminal offence. Society grants professionals such a monopoly in return for the provision of certain services.

However, this account of professional standards assumes that professional and legal obligations always overlap. This seems to suggest that the legal model of professional standard does not, in fact, yield any distinctively professional standard but only a legal framework for professionals. This is problematic. Conceptually, professional and legal expectations are different and independent of one another. There are cases in which it seems intuitive, and on which most people would agree, that acting as a professional implies acting against the law. For example, there have been cases of nurses refusing to force feed US political prisoners, which would violate the professional requirement to obtain informed consent.^16^ Even if one thinks that force feeding is morally or legally acceptable, one would probably agree that infringing on informed consent in this way violates at least the internal professional standards of medicine. In addition, acting within the law might mean acting unprofessionally. If a state prohibited the provision of basic healthcare to illegal immigrants, healthcare professionals would arguably retain a professional obligation to provide it.^17^

It is worth emphasising that we merely want to point at the *conceptual* difference between legal and professional standards and to challenge the assumption that professional obligations should automatically incorporate an obligation to respect the law. In the aforementioned statement, the AMA explicitly adds that doctors are being permitted to perform abortion ‘under circumstances that do not violate the law’, but this fails to recognise the conceptual distinction.

Thus, the legal model does not offer professional organisations any guidance as to what to do when professionals want to act against the law, but according to what would otherwise be professional standards.^1^ After all, these members of the profession may well be guided by a conscientious commitment to always act professionally.^18^

On some views, professional obligations are determined by the internal standards of the profession.^19^ However, there is an ambiguity in the notion of ‘internal standard. It might refer either to the requirements set out in the relevant professional codes of practice or to some internal ethics of the profession which is independent of such codes. Let us analyse the two options in order.

#### The ‘Professional Organisation’ model of professional standards

According to the ‘Professional Organisation’ model, what counts as a professional standard is established by the codes of practice of the relevant professional bodies, such as the Royal College of Obstetricians and Gynaecologists in the UK or the American College of Obstetricians and Gynaecology (ACOG) in the USA, to give just two examples. These are the standards one voluntarily accepts to abide by when deciding to enter the profession that is internally regulated by those bodies. As all codes, they can change over time or from one place to the other. Sometimes, individual members of the profession need to accept the existence of professional requirements they do not agree with or that were not present when they started their career—healthcare is hardly unique in this respect.

It can of course be debated how those professional standards should be decided in the first place. For instance, one could ask whether it should be members of the profession themselves (as has traditionally been the case) who self-referentially give themselves their own standards; or whether other types of experts and knowledge should be involved (eg, from ethics and the social sciences); whether patients should be involved in the construction of such internal morality of medicine^20^; or whether it should be a matter of expertise at all, as opposed to a process that involves society at large. Indeed, the mere fact that there currently are certain professional standards in place does not mean these standards are right or the procedures through which they have been formulated are appropriate. It seems one necessary aspect of such standards is that they should conform to basic standards of the ethics that is specific to the profession in question. In this case, medical ethics.

One problem with this approach is that it leaves professional organisations with a lot of discretionary power to decide what professionals may or should do, potentially at the cost of ethical requirements. For example, if medical codes contain the professional requirements to operate within the law, they risk incorporating any such violation that the law contains. Consider the following, well-known case.

Savita Halappanavar was a 31-year-old woman living in Ireland who was refused a uterine evacuation. Ms Halappanavar, who was 17 weeks pregnant, presented at University Hospital Galway and after it was determined that she was miscarrying, her repeated requests for a termination were refused. Despite the fact that the fetus would have died anyway, doctors insisted that, as long as there was a fetal heartbeat, *legally and professionally* they could not do anything. She died of overwhelming sepsis.^21^

This is a clear breach of an ethical duty to protect the best medical interests of the patient. This is plausibly true also in the view of someone who thinks abortion should, generally, not be part of medical practice and that the fetus counts as a patient. Since it was certain that the fetus would die, there can be no plausible ethical or professional reason to jeopardise the woman’s life, even if the fetus was believed to have some or full moral status.

Cases of abortion are normally more ethically problematic than this, of course, because often there is a genuine conflict between health interests or desires of women and the value of the life of a fetus who would otherwise survive. But the example here is merely meant to illustrate how the law can fall short of meeting uncontroversial standards of medical ethics. A professional code of practice requiring professionals to operate within the law would just incorporate the same problem.

To avoid this implication, we would need to say that, at the very least, being a professional means following the standard set by professional bodies, but only *provided they reflect proper standards of medical ethics*. This is because, plausibly, professionalism must mean more than just following arbitrary rules or rules that are not consistent with the ethics of the profession. After all, professional organisations have in the past permitted or silently accepted unethical practices, which were eventually corrected by legislation and by a change in professional guidelines. For instance, the German Medical Association accepted and indeed promoted many of the Nazi practices.^22^

Professional ethical standards and professional guidelines need to be aligned in order for professionalism to be a principle informing and *justifying* professionals’ conduct.

#### The ‘Best Medical Interest’ model of professional standard

The central professional obligation for doctors is to act in the best medical interests of their patients. There can be disagreement on what other standards there should be, who should be involved in the decision about it, or—crucially in the case of abortion—who counts as the patient whose interest should be prioritised. However, acting in a patient’s best medical interest is uncontroversially part of the proper scope of practice of healthcare professionals. It is perhaps useful to recall at this point why societies maintain professions in the first place. Professions are primarily maintained because an assumption is made that they best serve the interests of members of the collective. This explains why most health care professional codes of ethics mention in one form or another that the professionals’ obligation is to prioritise their patient’s health and well-being over other considerations. However, again, the fact that they typically do it does not mean that the two standards—Professional Organisation and Best Medical Interest—are conceptually the same.

Whether the Best Medical Interest model professionally justifies abortion provision in any specific case depends on whether there are risks for the health or the life of the woman if she does not obtain an abortion. This assessment depends on many factors, including a plausible understanding of physical and mental health and whether counterfactuals should be taken into account when assessing risks. For example, would failure to obtain an abortion undermine the woman’s health, according to some plausible understanding of ‘health’? Would the woman seek an illegal and potentially unsafe abortion which would put her health at risk? Would that make abortion provision in the best *medical* interest of the woman, as opposed to in her interest more broadly conceived? Would her mental health be compromised by having a baby she does not want? We leave the question open as to whether and how these factors should be considered in determining the best medical interest. For the purpose of this paper, we simply point out that the Best Medical Interest model implies that, as far as the woman is concerned, abortion provision is consistent with professional standards informed by good medical ethics when it is in the best medical interest of a woman.

This might be difficult to establish in certain cases. Some would say that in every case in which a woman requests an abortion, the abortion is in her best medical interest because the counterfactuals mentioned above matter for the purpose of determining the best medical interest. Others would say that it is only in a woman’s best medical interest when not receiving an abortion would result in some physical or mental health impairment for the woman because of the continuation of the pregnancy. Addressing these issues requires providing a definition of health that allows to determine whether a woman’s health can be said to be negatively affected by the failure to obtain a safe abortion. Here, we just want to point out that according to this criterion, this is the kind of issue that would need to be settled by professional organisations to determine when abortion provision falls within the proper scope of professional practice.

Many, including many medical professionals and some sectarian professional bodies (such as the Catholic Medical Association), believe that the best medical interest of the fetus should be factored in. Indeed, the US States that will prohibit abortion will likely do so precisely because many people living there believe that a fetus has moral status that warrants protection of the fetus’ interests and rights.

Now, the moral status of the fetus is a contested issue, e. How much priority should women have over foetuses, when it is not their life to be at stake, but their health or perhaps just their preference? This is a difficult question to answer. However, on the assumption that it is at least *reasonable* to think that foetuses do not have a moral status that outweighs the value of women’s health and autonomy, the ‘best medical interest’ criterion implies that at very least doctors will be professionally *justified* in providing abortions to women in jurisdictions where it is illegal, when there is a serious threat either to their life or to their health. After all, they are acting on an ethical view, that is, by hypothesis, reasonable according to the ethical standards of the profession. Whether they are also professionally required depends on whether stronger ethical claims about the lack of moral status of foetuses and, therefore, their status as patient can be made, but we will not discuss this here.

### Best Medical Interest and Professional Organisation models: jointly necessary and sufficient for professional obligations?

Ideally, Professional Organisation model and Best Medical Interest model would overlap, in the sense that ideally we would want professional codes of practice to be informed by the best medical ethics standards. In fact, they often do overlap. However, because they are conceptually distinct, they raise the question of whether they are jointly necessary and sufficient for grounding professional obligations, or whether either is by itself necessary and sufficient.

The Best Medical Interest model, if adopted, would be sufficient for the professional *permissibility* of breaking the law in cases in which there is reasonable disagreement on the ethical aspects of a certain procedure that are relevant to medical ethics (eg, if the fetus has the moral status of a patient). This might or might not be all-things-considered ethical and might legitimately subject the professional to legal sanctions, as we shall discuss shortly. It is also sufficient for a professional obligation in cases in which there is no reasonable disagreement about the morality of the procedure in question from the point of view of medical ethics (say, providing healthcare to illegal immigrants). In principle, it is also sufficient to justify a professional *obligation* to act in the best medical interest of the patient.

In practice, however, we see a danger in relying exclusively on the Best Medical Interest model as it gives individual practitioners significant discretionary power in deciding what counts as in the patient’s best medical interest, as the example of sterilisation mentioned above suggests.

Embedding the ethical principles in codes of practice would offer more solid grounds for professional obligations, as codes of practices can be subjected to public scrutiny and revisions in light or better arguments and evidence. The Professional Organisation model informed by the Best Medical Interest model, that is, professional codes informed by good medical ethics, provide a more objective criterion for clinical decision-making that is less susceptible to arbitrary use during clinical encounters.

In the case of abortion, at the moment, most non-sectarian codes of practice consider it consistent with good medical practice and with medical ethics. Moreover, we have seen that it is at least reasonable to assume that foetuses lack the kind of moral status to warrant their status as patient. This means that, at the moment, the Professional Organisation model informed by the Best Medical Interest model implies that it is at least professionally permissible for a healthcare professional to provide, in certain circumstances, illegal abortions. All of this might well be questioned, of course, and should constantly be kept under scrutiny. People who disagree with it should be free to lobby and persuade relevant professional bodies to change this position. If this stance is found wrong, professional guidelines should change to reflect the fact that a fetus has the full status of a patient when a woman presents herself to request an abortion. In that case, abortion might well have to be ruled out as a matter of professional obligation, regardless of whether it is permitted or prohibited as a legal matter. However, as things stand now, professional organisations’ codes recognise the woman as the primary patient, which is consistent with a plausible understanding of the Best Medical Interest standard of professional obligations based on reasonable, defensible ethical arguments.

One problem here is that there seem to be quite a few cases in which pursuing the best medical interest of a patient would require contravening other ethical or societal goals or other non-medical interests of the patient. One example is male circumcision, which is often undergone for cultural reasons and might be consistent with an ethical principle of respect for cultural or religious traditions. Male circumcision is legal in most countries. However, it is not necessarily in a patient’s best medical interest. Similarly, the prohibition of abortion might reflect the cultural values of a certain community and be part of its tradition. This type of cases is a good reminder of how, consistently with the distinction we drew at the beginning, ethics is broader than just medical ethics. Values of the latter might be in tension with the former. The healthcare profession is not concerned with the promotion of cultural traditions, but one might defend the ethical value of promoting cultural traditions in the political arena, outside of the healthcare context. These are cases of genuine conflict between strictly professional and broadly ethical demands. Here, we are only concerned with what one ought to do as a healthcare professional, that is pursue patients’ best medical interest, at least when it is consistent with other professional and ethical obligations (say, fair allocation of resources). It might well be that sometimes professional obligations require professionals to act against broader ethical values, in the same way as they sometimes require to act against the law.

### Acts, omissions and professional settings

As Lisa Harris pointed out 10 years before the reversal of *Roe v Wade*, while conscientious objection is typically understood in terms of refusal, the *provision* of abortion care can also be a matter of conscience. The question arises as to how conscientious commitment compares, ethically, to conscientious refusal to provision of abortion services. After all, we are in both cases talking of a conscientious objection to what would otherwise be a legal or professional expectation. It is important to focus on this aspect of abortion provision, especially where legal access to abortion is constrained, because, as Harris says,

The persistent failure to recognize abortion provision as “conscientious” has resulted in laws that do not protect caregivers who are compelled by conscience to provide abortion services, contributes to the ongoing stigmatization of abortion providers, and leaves theoretical and practical blind spots in bioethics with respect to positive claims of conscience^23^

The reversal of *Roe v Wade* has created a situation where positive claims of conscience could be more frequently put forward. As sociologist Carole Joffe pointed out, especially when abortion was illegal, abortion provision often was a matter of conscience. It was grounded in doctors’ deeply held moral beliefs that ‘abortion provision honoured “the dignity of humanity” and was the right—even righteous—thing to do’.^24^ The reversal of *Roe v Wade* might make these reasons of conscience salient again in a doctor’s decision whether to provide an illegal abortion. Elizabeth Sepper and Dov Fox have argued that if conscience has to be taken seriously within the law, conscientious commitment should be protected at least in legislations that protect negative claims in the attempt to balance interests of institutions, professionals and patients. This means, for example, that practitioners should enjoy legal protection when they operate within institutions that do not provide a certain service (like abortion), but conscientiously decide to provide it.^25 26^ These authors are concerned with legal accommodation of positive claims of conscience. However, at the moment positive claims do not enjoy legal protection. This is why here we are concerned with how professional organisations should regulate the matter in their professional guidelines and codes of practice, given the lack of legal protection.

If practitioners have a professional obligation to provide in certain cases illegal abortion or at least they are professionally justified in doing so, then professionals with a conscientious commitment to abortion provision would find adequate ethical or professional reasons to back their conscientious commitments. The approach of professional organisations should reflect this ethical framework.

The debate on conscientious objection in healthcare has mostly focused on negative claims of conscience, i.e. conscientious *refusal*.^16 27–32^ Discussion of positive claims of conscience, or conscientious commitment, is less frequent and less developed. It has typically focused on either the possible legal accommodation of positive claims^25 26^ or on broader ethical issues around the ethical relevance of the act-omission distinction.^23 33–39^ As we said, here we are focused primarily on how professional organisations should treat positive claims of conscience to provide illegal abortion. However, to address this question, it is necessary first to look at what role the act-omission distinction plays in grounding professional obligations.

Some appeal to the moral relevance of the act-omission distinction to claim that negative claims of conscience are easier to ethically justify than positive claims.^38^ We can call this the ‘asymmetry thesis’. Others defend the asymmetry thesis by appealing to the moral asymmetry between positive and negative *rights*, rather than positive or negative claims. According to this view, positive claims of conscience would often need to be treated as positive rights. That is, they would require institutions to actively take steps and make special arrangements to ensure that procedures that are otherwise prohibited can be carried out by the doctor with the positive claim. In contrast, negative claims of conscience would give rise to a negative right, that is, they would not require actively putting in place any special arrangement to be accommodated.^2^ 34

There are two ways to respond to these claims.

Starting from the latter, it is simply not true that the right that negative claims would give rise to is negative in the sense just explained. If an institution is committed to providing abortion, or is required to provide abortion as a condition for receiving state funding, then accommodating conscientious refusal might require institutions to take action and use resources to ensure that abortion services are available (eg, by hiring more staff willing to provide abortions).^35^

With regard to the former claim, the relevance of the distinction between negative and positive claims of conscience within the healthcare profession can be questioned.^35–37^ The asymmetry between negative and positive claims does not apply to the healthcare context in the same way as it applies to other contexts. Normally, ethical values and principles ground professional standards that are less sensitive to ordinary moral distinctions, or indeed not sensitive at all. Rather than transferring ordinary moral distinction into professional settings, we normally consider professional settings as particular areas where ordinary moral distinctions are replaced by professional standards. For example, professionals like firefighters or police officers are not held accountable on the basis of the act-omission distinction in the same way as most of us are in our everyday life. They have professional obligations to take action for the sake of people in danger when most of us do not have corresponding ethical obligations to do the same.

Indeed, in the case of abortion, according to the Best Medical Interest model, the claims of conscientious commitment might even be stronger than claims of conscientious refusal because in the case of commitment doctors are responding to the autonomous request of a being with clear interests. This is not the case when considering conscientious objection to the provision of abortion, given the uncertainty and reasonable disagreement around the moral status and the interests of the fetus. Thus, the claims of refusers seem weaker. One could plausibly argue that conscientious refusal could be granted without it resulting in significantly suboptimal care for women. However, the chances that objections result in suboptimal care seem high. In fact, there is significant evidence suggesting that conscientious objection does create significant risks of harm to women.^40 41^ Besides, unequal distribution of the burdens of abortion provision among practitioners might create imbalances, resulting in suboptimal care, for example, if there are few doctors that have to provide all the abortions (which also raises an issue around fair working conditions, which we will not discuss here).

Now, even assuming that the act omission-distinction does not play any relevant role in the comparison of conscientious refusal and conscientious commitment in healthcare delivery, there are other factors to consider when asking how doctors should or may handle requests for illegal abortions. We need to distinguish between professional obligations/permissibility, ethical obligations/permissibility and legal protections of conscientious abortion provision. Let us consider all these in order.

### Positive claim of conscience as a professional obligation/professionally permissible?

If we adopt the first criterion and we think that healthcare professionals have a professional obligation to provide *all and only* the services for which their patients are *legally* eligible, it follows that doctors have also a professional (and not just a legal) obligation not to provide illegal abortions, even when they think abortion is consistent with good medical practice as defined by professional standards of relevant medical organisations. In this view, the reversal of *Roe v Wade* means that healthcare professionals operating in states that legally ban abortion have a professional obligation not to provide it to women who request it, even when they think abortion would otherwise meet professional standards. Indeed, in some countries including Nicaragua, Chile, El Salvador and Malta, even when women’s life is at risk. This view is implausible, as the case of Mrs Halappanavar demonstrates. Even if illegal, it seems plausible that healthcare professionals had at the very least professional ethical licence to save Mrs Halappanavar’s life by terminating a pregnancy that would anyway have naturally ended with the death of the fetus.

If we instead adopt the Professional Organisation model informed by the Best Medical Interest model, things get more complicated. If abortion provision is consistent with good medical practice as determined by relevant professional bodies *and* consistent with the promotion of a patient’s best medical interest, then doctors might well have a professional obligation to provide it. That is, it might well be the case that healthcare professionals sometimes have *professional* obligations to break the law—after all, this was the case with doctors practising in Nazi Germany who were required by law to deny Jews appropriate medical care. Or, to put it differently, it might well be that respecting the law would sometimes require them to fall short of meeting their professional obligations. However, given the uncertainty and reasonable disagreement around the moral status of foetuses and the fetus’ interest, it is plausible to say that the combined model grounds at least the professional *permissibility* of providing, in certain cases, illegal abortions.

### Positive claims of conscience as an ethical obligation/ethically permissible?

Suppose we accept that healthcare professionals do not always have a professional obligation to obey the law. Instead, they have a professional obligation to stick to the standards of good medical care, as determined by good medical ethics and the relevant professional codes. The next question is whether they also have an all-things-considered ethical obligation to follow the law or to follow professional obligations. Arguably, breaking the law for the sake of sticking to professional obligations might be too demanding to be considered an ethical obligation, at least if we accept that supererogatory acts—while they might be praiseworthy—cannot be a matter of ethical obligation. Fulfilling a professional obligation might land doctors in jail. It is, therefore, plausible to say that doctors do not necessarily have an ethical obligation to provide illegal abortions, even assuming they have a professional obligation to do so. Besides, most obviously, even granting that there is a professional obligation to break the law at times, many would say that the ethical obligation to abide by the law is stronger independently of considerations of the personal costs that breaking the law implies, which leads to the next question: Could it at least be ethically *permissible* for doctors to provide abortions in such cases?

That acting against the law is (at least) ethically permissible is, needless to say, a problematic claim. We can safely assume, at least in liberal democratic societies, that there is a *prima facie* obligation to respect the law. But what if a law is unethical, as was arguably the case in Ireland where the preventable, unnecessary death of Mrs Halappanavar was the consequence of doctors simply following the law? It seems quite plausible to claim that to act against an unethical law is, at times, ethically permissible. Of course, this raises the question of when abortion prohibition is all-things considered unethical, which we are here happy to leave open as it might include considerations that extend beyond strictly *medical* ethics.

### Should positive claims of conscience be granted some legal protection?

Typically, conscience clauses allowing conscientious refusal in laws that legalise or decriminalise abortion do not undermine the *legal* availability of the procedure—although they could undermine the availability *in practice*.^30^ Thus, conscientious refusal to provide abortion can be legally consistent with the legalisation of abortion.

However, in the case of positive claims of conscience, the distinction has more problematic implications. Conscience clauses allowing abortion provision in laws that prohibit abortion would defeat the purpose of the legal prohibition. Quite simply, they would entail that what is prohibited could sometimes be done, that is, that it is in fact not prohibited.

To be sure, there have been cases where police and prosecutors had discretionary powers and chose not to prosecute offenders, when they believed that the harm caused by prosecution and sentencing would be greater than the harm of the offence that was committed. For example, for many years in the Netherlands cannabis use was illegal, but offenders were not prosecuted precisely for this reason. Prosecutorial charging guidelines taking this into account in the case of enforcement of restrictive abortion laws might be a realistic possibility in some communities. However, other societies are stricter in the way they enforce laws, in the sense that the damage of law enforcement is not taken into consideration or is considered not to outweigh the benefits of law enforcement.

The application of the law often takes into account mitigating circumstances for those that act against it. This is where there is some scope for offering *at least some* protection to doctors who provide abortion consistently with professional organisations’ guidelines and in the best medical interest of women.

Some have suggested that a legal system based on solid ethical principles would give more protection to positive claims of conscience and less protection to negative claims of conscience. This is because, among other reasons, positive claims honour patients’ requests and conscientious refusals override them.^26^ Our arguments concern professional obligations and guidelines and not legal reforms. We are, therefore, not committing to any such claim here. However, our argument on the importance of promoting patients’ best medical interest provides further support to these legal arguments.

## Conclusion

Professional organisations often include in their professional codes of practice the professional requirement to operate within the law. This means that a practitioner who acts against the law, but according to what would otherwise be professional standards (eg, beneficence), is acting unprofessionally and, therefore, could lose their licence. For example, we have seen at the beginning that the ACOG has adopted a very strong stance against antiabortion laws. However, the ACOG’s Code of Professional Ethics is very explicit in stating that ‘[a]s a member of society, the obstetrician–gynaecologist should respect the laws of that society’.^42^

One practical upshot of our discussion is that, *if* we think abortion is in the best medical interest of a woman and considerations of women’s interests trump considerations of a fetus’ moral status, then professional codes should remove the explicit requirement to operate within the law in their guidelines on abortion. Also, professional organisations should not punish their members and should indeed lobby to protect them against legal sanctions that pertain to the professional sphere, such as the deregistration of professionals that adhere to good standards of medical ethics. After all, they were following professional standards, which is the only thing that falls within the area of competence of professional organisations. On this view, if a practitioner decides to follow a professional standard and break the law and incurs legal punishments, the practitioner would be able to practice again once those punishments have been administered.

While the law will have the greatest power to influence clinical practice, professional organisations still retain considerable authority and power over their members. They can choose to deregister, suspend or place constraints on practice of their members, even when the law does not require it. They can choose to readmit members after legal punishments have been served. They can lobby courts or judges to reduce sentences, or advise against deregistration. An active and ethical profession can have a significant influence over the law. For example, for years, resistance by medical organisations has hampered the passing of legislation to allow assistance in dying, despite widespread public support.

Some might want to push this line of argument even further and claim that professional organisations should encourage civil disobedience by their members.^43^ Civil disobedience, unlike conscientious objection, is a political act involving deliberate breaking of the law aimed at bringing about changes in legislations considered unjust. Unlike conscientious objection, civil disobedience comes with the acceptance of the legal penalties for breaking the law. Civil disobedience raises many other ethical and political issues that are beyond the scope of this paper. Most notably, respecting laws one does not agree with is important in order to maintain an orderly, civil society. In civil disobedience, the primary intention is to change the law; in the case of conscientious provision of medical care, the primary intention is to promote the best medical interests of the patient. While there might well be overlapping between the two, here we are simply claiming that professional organisations should protect as much as they can their members that operate in their patients’ best medical interest, regardless of whether they act also qualifies as civil disobedience.

When a law regulates a professional practice that is considered consistent with professional standards, like abortion, the potential for conflict should be acknowledged by the relevant professional organisations. To simply claim that professionals should act within the terms of the law is to refuse to acknowledge the possibility of a conflict between standards of medical ethics and the law. As we argued above, this is a mistake. The role of professional organisations is that of regulating professional conduct, not to enforce the law. Although the law trumps professional standards in case of conflict and those breaking the law should be prepared to pay the legal consequences, the application of the law falls within the competence and responsibilities of relevant state authorities.

The Medical Council of Ireland, where Mrs Halappanavar’s case occurred before abortion was legalised, currently states under the heading of ‘professionalism’ that ‘doctors must always be guided by their primary responsibility to act in the best interests of their patients, without being influenced by any personal consideration. They should act independently in the service of their patients and have a responsibility to advocate with the relevant authorities for appropriate healthcare resources and facilities’.^44^ Our argument implies that this should be the sole standard for professionalism of healthcare professional organisations, including in dealing with illegal abortions.

## Data Availability

No data are available.

## Footnotes

The legal model does not raise these problems in the case of negative claims of conscience because typically the procedures doctors conscientiously object to are (considered to be) within professional standards, and therefore there is no conflict between the law and professional standards. Thus, the legal model could be used to argue against conscientious objection to provision of medical services, although we are not addressing that issue here.

One might note that positive conscience claims can be in opposition to the law as well as in opposition to hospital policies, where the contested procedure might not be illegal in the local jurisdiction. We are only concerned in this paper with the former situation.
